# The Factors Influencing Seasonal Dynamics and Spatial Distribution of Stable Fly *Stomoxys calcitrans* (Diptera, Muscidae) within Stables

**DOI:** 10.3390/insects9040142

**Published:** 2018-10-16

**Authors:** Marek Semelbauer, Barbara Mangová, Marek Barta, Milan Kozánek

**Affiliations:** 1 Institute of Zoology, Slovak Academy of Sciences, Dúbravská cesta 9, 845 06 Bratislava, Slovakia; uzaebama@savba.sk; 2 Institute of Forest Ecology, Slovak Academy of Sciences, Ľ. Štúra 2, 960 53 Zvolen, Slovakia; Marek.Barta@savba.sk; 3 Scientica s.r.o., Hybešova 33, 831 06 Bratislava, Slovakia; milan@scientica.sk

**Keywords:** pest control, cattle, confined facilities

## Abstract

The biology of the stable fly is fairly well known, but factors influencing the distribution of adult stable flies within stables are still inadequately investigated. The four experimental stables were located in south western Slovakia. Within each stable, five sticky traps were localized along the stable, and the flies were weekly counted during the flight season of years 2015–2017. Seasonal activity and stable fly abundance in relation to temperature, rainfall, light conditions, relative air humidity, and cows per stable were evaluated. The seasonal activity of the stable fly shows one large peak at the end of summer and a second smaller peak just before the end of the flight season. The spatial distribution of stable flies was unique for each stable. All of the environmental variables had significant and mostly positive effect on stable fly abundance. The strongest and most positive effect on stable fly counts was temperature and rainfall five weeks prior to collecting session. Within the stable, cow number, air humidity, and light conditions are the strongest candidates to influence their distribution.

## 1. Introduction

Stable fly, *Stomoxys calcitrans* L., is a well-known major pest of confined and pastured cattle. Stable flies cause animals discomfort due to their painful stabbing and ability to transmit pathogenic microorganisms [1,2]. Animals react to fly attacks by foot stomping, head throwing, skin twitching, and tail switching; Mullens et al. 2006 [3], The irritated cattle graze for a shorter time, and cattle frequently bunch together. The bunching behavior leads to increased body temperatures and lower milk production [4,5]. Blood losses due to the stable fly feeding result in reduced gain in weight and reduced milk production [4]. Reported infestation levels range from 2 to 24 flies per leg [4], but Solórzano et al. [6] report as much as 700 flies per animal. More than three stable flies per leg can cause economic damage [4,7]. The corresponding estimated annual loss of milk production per cow can range from 42 to 299 kg, with a median of 139 kg. Economic losses in 2009 dollars have been between $13 and $85 per cow per year in the United States [4].

The biology of the stable fly is fairly well known. Larvae are saprophagous, living in moist decaying vegetable matter (such as silage, decaying hay, or lawn cuttings). Cattle manure alone is not a proper substrate for larval development unless it is mixed with vegetation [4]. The complete cycle can take only three weeks in optimal conditions. Both sexes suck blood, though when in the migratory phase, they visit flowers [7]. Adults prefer to feed on the cow’s front legs and the preferred time for feeding is early morning or late evening. Females need 1.8 feedings per day while males averaged 2.8 feedings per day [8]. Single females can produce 60–800 eggs during their lifetime [9]. From environmental factors, the rainfall and temperature are known to influence the population dynamics of stable flies [4,10,11]. Models have been developed that allow for predicting stable fly abundance based on environmental data [12,13]. Fly movement in dairies during spring and summer is usually limited. However, the stable flies are capable of flying almost 30 km within a day [14] and up to 225 km over several days [15].

Management options to control stable fly population include removal of potential breeding sites, insecticide treatments targeted directly on cows, residual/contact pesticides applied near fly resting (walls, ceilings) or breeding sites, insecticide-impregnated ear tags, various fly traps (alsynite trap, stablebands), applying parasitoid wasps attacking pupae (*Spalangia* and *Muscidifurax* spp.), and entomopathogenic fungi infecting adults or larvae [7,16,17]. Aside from the parasitic wasps and ear-tags, the question arises as to where to apply the insecticide or localize the trap so that as many flies as possible will be hit. Although local and long-range dispersal of stable fly have been reviewed recently [18], little is known about factors influencing the distribution of stable flies within cattle facilities.

The aim of the present paper is to study factors ruling the stable fly spatial, as well as seasonal, flight activity in confined livestock facilities in temperate climatic conditions of central Europe.

## 2. Material and Methods

### 2.1. Study Site

The field experiments were conducted at the cooperative farm PD Šenkvice located in the village of Šenkvice. The cattle farm is situated in close vicinity to the village (48°18′10″ N, 17°21′34″ E, altitude 177–178 m above sea level) in the foothills of the Little Carpathian Mountains. The climatic conditions in this part of Slovakia are characterized by warm summer (mean July temperatures: 18 to 21 °C) and cold winters (mean January temperatures: −1 to −2 °C). Annual precipitation reaches 500 mm and most of the rain falls during spring and autumn. In combination with high summer temperatures, this results in dry summers.

### 2.2. Experimental Stables

**Figure 1:**
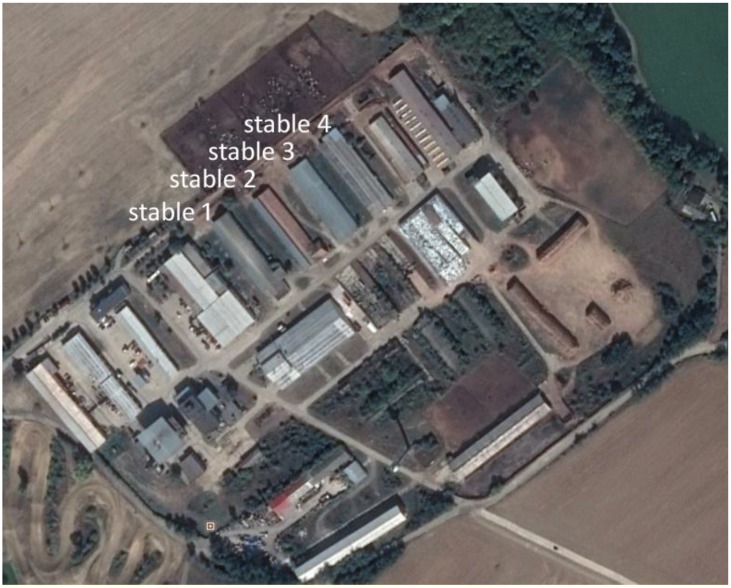
Area of the farm with four study stables. Several more stables are present, but were not included in the study. North of the stables, a fenced area is visible, where the animals stay while the stables are cleaned. Source: Google Earth.

The farm consists of six stables arranged in parallel in a southeastern (entrance) to northwestern (exit) direction. A complex of four stables neighboring each other was selected to accomplish the experiments. Each stable was intended to house cows in different stages of the milking and reproductive cycle. Information provided by the farm staff was as follows: stable 1–60 low milking and gravid cows; stable 2–56 highly milking and gravid cows; stable 3–56 highly milking, gravid, and inseminated cows; stable 4–56 highly milking, gravid, and inseminated cows. The internal stable dimensions were 70 × 10 meters (Figure 1).

### 2.3. Collecting Device

The device used for insect collection was made of a stainless steel holder and a sticky board. Each holder consisted of anchor, arm, and clip. The anchor of the holder was fixed on the wall at a height of 150 cm. The 30 cm-long arm prevents contact of the sticky board with the wall. The yellow plastic sticky board of size 20 × 30 cm was joined to the holder by the clip on its outer end. Plastic Polyvinil chloride sticky boards were obtained from Papírna Moudrý, Ltd. (Židlochovice, Czech Republic)—an industrial producer of plastic sticky boards for the control of cherry fruit fly *Rhagoletis cerasi*. Initially, white, yellow, and black sticky boards were tested prior to the experiments, the collecting results were the best with yellow boards. The plastic boards were replaced weekly. One set of holder and sticky board represented one collecting point.

### 2.4. Data Collection 

Five collecting points were distributed inside each experimental stable. The first and the last collecting points were located 10 m from the entrance and the exit of the stable, respectively. The collecting points were deployed at regular 15 m intervals. The collecting season started in the first week of May and ended in the last week of November; farm inspections were performed weekly. The front and the back sides of the sticky board of each collecting point were photographed by Nikon D700 and AF-S NIKKOR 24–70 mm. The stable flies were counted from each photo in the laboratory. Specific body shapes of stable flies, their long proboscis, and a high photographic resolution allowed magnification of the pictures via computer to assure reliable determination.

Climatic data (mean week temperatures, total rainfall per week) were obtained from the Slovak Hydrometeorological Institute and originated from a meteorological station Kráľová pri Senci, situated approximately 10 km from the farm. Light intensity data were measured by Omega HHLM-1 device. To provide more insight into the distribution of stable flies within stables, in the year 2017, we employed the relative humidity measured by dataloggers (EBI 20-T/-T1/-TE/-TE1 and UX100 Temp/RH) mounted on the arm of each stick trap. During the year 2017, the exact number of cows present in each stable was also counted at each collecting session.

### 2.5. Data Analysis

Data were analyzed in R, version 3.0.2 [19]. Packages nlme [20] and lattice [21] were used for the analysis and as graphical tools. As explaining variables, the following were used: mean temperature in the collecting week, precipitation in the collecting week and precipitation during one to six weeks preceding the data collecting week, surface of the stabled and exposed, quadratic (precipitation and temperature), and one interaction term (precipitation/temperature).

Prior to analysis, the explaining variables were checked for collinearity by pairs plot. The stable fly counts were log transformed to allow for the use of the models assuming Gaussian distribution of the residuals. Further analysis followed general protocol described in detail by the authors of [22]. First, a null model was fitted with gls. As there are multiple sources of correlation in our data (position within stable, within stable, within year, time correlation along the season), several correlation structures were introduced in the model. The best model was selected based on the AIC (Akaike information criterion) and compared against the null model. The significant difference between the models was considered as strong support in the presence of correlation in our dataset. Once the optimal variance structure was found, the optimal fixed structure was found by backward selection, that is, non-significant terms were dropped (one each time) and compared against the original model by likelihood-ratio test. Non-significant difference between the models resulted in acceptance of the simpler model. Once all of the terms were significant, the backward selection was stopped.

## 3. Results

### 3.1. Climatic Conditions

**Figure 2:**
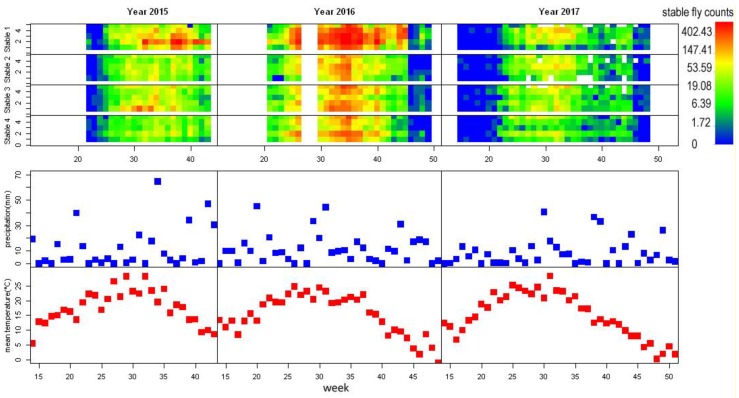
From top: Stable fly counts per sticky board and week in stables stable 1, stable 2, stable 3, stable 4 precipitation rate per week (in mm), and mean week temperature (in °C). The log-transformed stable fly counts are expressed in colour, white spaces represent missing data; x axis represents time in weeks, for stable fly counts, the y axis represents position within stable. Note the low precipitation rate in year 2017 and corresponding low stable fly traps.

The years can be ordered according to the rainfall from driest to wettest: 2017 (415 mm), 2015 (505 mm), and 2016 (550 mm). Differences in the rainfall among the years were substantial. The rain falling in the first three months was almost double in years 2015 and 2016 compared with that in 2017. On the other hand, there was no distinctive difference in the temperature between the years (Figure 2).

### 3.2. Stable Flies Abundance and Flight Season

A total of 90,479 stable flies were trapped throughout all three seasons. The maximum number of stable flies trapped on a single sheet was 824. The three years differed considerably in the number of stable flies counted (2015: 20,459, 2016: 58,899, 2017: 11,121). The flight season of stable flies lasted from the second half of May to the end of October, with peak abundance at the end of August and in September. A second smaller peak might appear just before the end of the flight season (Figure 2).

### 3.3. Distribution of Stable Flies among and within Stables

The four study stables differed considerably in number of captured stable flies. Stable 1 obviously had more stable flies than the other stables, and peak numbers of stable flies on traps in stable 1 were almost two-fold higher than the other three stables (Figure 2). The actual count of stable flies could be even higher in stable 1 compared with the remaining stables if the size of the sticky board (20 × 30 cm) was larger. When the sticky surface of sheet is completely covered by flies, it can no longer trap flies.

The distribution of stable flies captured within stables followed a unique pattern for each stable. However, it was surprisingly consistent throughout the years, for example, in stable 4, trap positions 2 and 5 always captured the highest number of stable flies (Figure 2). The fly abundance was fairly synchronized across stables and positions within stables, although some positions caught disproportionally high numbers of stable flies (e.g., position 2 in stable 1, year 2015, Figure 2).

### 3.4. Influence of Environmental Factors on Numbers of Stable Flies Captured within Stables

Pairs plotting suggested collinearity between precipitation in week 0 and week 1 before the collecting week, thus the latter variable was excluded after finding the optimal variance structure (exponential correlation in four dimensional space; dimensions were defined by year, week, stable, and position within the stable). From the tested factors, only I (precip^2) term could be dropped by backward selection. Dropping of all other factors caused significant worsening of the model (increase in AIC). The results suggest that precipitation and temperature, as well as light conditions, have positive effects on the stable fly occurrence in stables (Table 1 and Table 2).

### 3.5. Influence of Microclimate on Numbers of Stable Flies Captured

**Figure 3:**
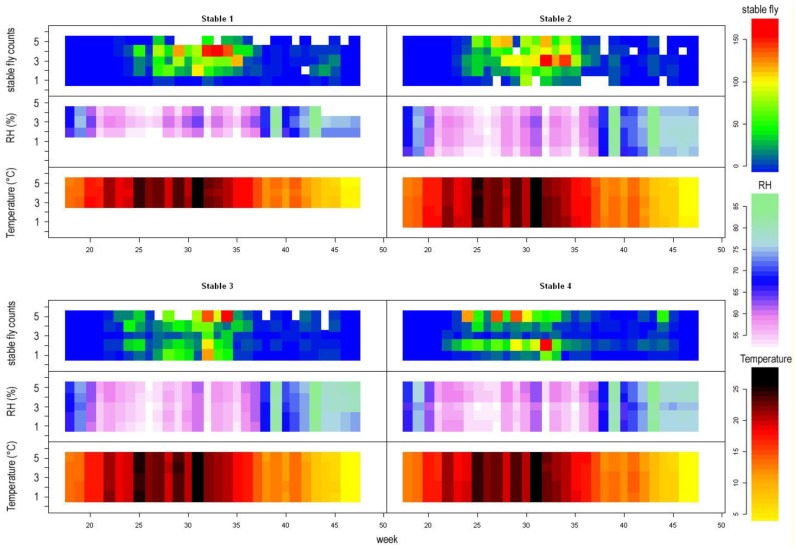
Stable fly counts, mean relative humidity (RH, in mm), and mean temperature (in °C) as recorded by datalogers from the year 2017; x axes represent time in weeks, y axes represent position within the stable (1–5). White space in stable fly counts represents missing data. Note that peak stable fly counts occur in weeks with low air humudity (white to purple, e.g., week 32).

To provide more detail on distribution of stable flies within stables, we analyzed stable flies captured from the year 2017 with additional environmental variables including (in addition to the previous ones) counts of cows per stable and mean, maximal, and minimal ambient temperature. The relative air humidity and temperature within the stables were recorded by datalogers. Unfortunately, from stable 1, there are missing data from positions 1 and 2. Stables 1 and 4 appear to be more humid than stables 3 and 4, but at least in stable 1, this might be caused by missing data from sticky traps located close to the entrance. The relative humidity as well as temperature change along the stables. Most humid positions appear to be either near the exit or in the centre of stable. Temperature shows a reverse trend. This is in accordance with the position of stables, where the entrance faces south-east, while the exit faces north-west (Figure 3). Pairs plotting revealed serious collinearity between the minimal, maximal, and mean temperature and temperature within stables; consequently, only the temperature within stables was retained. Similarly, mean relative humidity was collinear with either minimal or maximal relative humidity in the respective week, and was dropped. As in the previous case, several correlation structures were introduced in the model and compared with the null model (without correlation structure). However, the best models according to AIC showed patterns in the distribution of the residuals; therefore, only models with an even spread of residuals were evaluated. After finding the optimal variance structure (Gaussian correlation in three dimensional space), the optimal fixed structure was found by backward selection. The results of the model are summarised in Table 3 and Table 4.

The second model is generally similar to the first one. The rainfall before the collecting date had a significant influence on stable fly counts, as well as the mean temperature. In contrast, the light condition had no significant effect in the second analysis. Most notably, the number of cows per stable and relative humidity have significant effects on stable fly abundance.

## 4. Discussion

### 4.1. Influence of Climatic Factors on Stable Fly Trap Catches

The seasonal trap catches of stable flies are strongly determined by the climatic conditions. The temperature and rainfall seem to be the most important. The flight activity of stable flies has a bimodal pattern in warmer regions (south western France, eastern Nebraska, Mexico; [12,23,24]), while a unimodal pattern is typical of tropical [25,26] and northern countries [13,27]. The unimodal flight pattern in the tropics is linked to the alternating of dry and humid seasons, while in the colder climate, it is regulated by alternating cold and warm seasons. In moderately warm (Mediterranean) or semiarid climates with dry summers and humid winters, the stable fly population typically has a bimodal pattern with peaks in spring and autumn. The supposed seasonal flight activity of stable flies in south-western Slovakia shows an intermediate pattern with a first large peak in summer and a second smaller peak in autumn. 

The developmental rate of the stable fly is highest in 30–35 °C (in laboratory conditions), but survival rate is highest in lower temperatures (20–30 °C) [10]. High summer temperatures should thus lead to a drop in stable fly population due to lower survival of larvae and pupae. A temperature of 35 °C also decreases the time of survival of adult flies of less than six days to 50% and decreases female fecundity. The net reproductive rate is highest in 25–27.8 °C [Lysyk, 1998]. This seems to be in accordance with our results, when the drop in stable fly catches occurs in September, following the relatively dry and warm July and August. It must be pointed out that air temperature and temperature of substrate, where the larvae breed, might be different. 

On the other hand, high rainfall should lead to an increase in stable fly population on farms and presumably to higher fly captures. Larvae of stable flies typically develop in moist vegetable debris, a habitat expanded by rain. In contrast, in some studies, the effect of rainfall on stable fly density was not significant (e.g., [18]), but this can be easily explained. Larval developmental time of stable flies is lasting about 2–3 weeks in optimal temperatures [7,28]. Therefore, the fly populations on farms should be linked with rainfall two or more weeks before the emergence of flies. In our study, rainfall in weeks preceding the collecting week, as well as rainfall during the week the data were collected, have significant and positive effects on stable fly abundance. Rainfall in weeks preceding the collecting week had a positive effect possibly because of enhanced larval survival (one to three weeks before the collecting week) and creating more larval habitat (three to six weeks before the collecting week).

### 4.2. Distribution of Stable Flies within and between Stables

It may be disputed whether fly catches on sticky boards reflect the true spatial distribution of flies or whether it reflects their activity in certain areas of the stable. The difference between these two options might be slight, but it is need to be aware of it.

Outside the stable, stable flies tend to rest on east and south facing surfaces [27], while they avoid windy conditions. Broce et al. [29] reported that stable flies prefer landing (on Alsynite cylinder trap) in places most protected from the wind and on the lower parts of the trap. The proportion of stable flies landing on the lower part of the trap increased with the wind speed. Stable flies also prefer lower perching sites (91% of flies were caught between 0.3–1.2 m by sticky traps) [30,31]. Stable flies thus seem to prefer sunny but sheltered sites, typically close to the ground. The behavior of the stable fly is also strongly influenced by temperature. When the temperature exceeds 30 °C, the stable fly is more active on the shady part of the host or moves to shelter. Typical resting sites during hot summer days may be trees or man-made structures, such as stables [18].

In the first two years, we noted the first stable hosted markedly more stable flies than the latter ones. The stables are roughly equal in size, but they differed by number of cows per stable. This was supported by our second model, where the exclusion of number of cows from the explaining variables caused increase in the AIC (worsening of the model). Throughout the season, the number of cows counted per stable changed constantly. Unfortunately, we lack cow counts from the first two years and thus we lost a potentially very important factor in explaining stable fly catches.

The distribution of stable flies within stables is far from random, according to our results. Certain positions within stables were more likely to catch a number of flies across the years. This suggests that there are some general governing factors that influence the occurrence of stable flies within stables. A possibly key is the behavior of cows, as stable flies stay in areas in the field where cattle will congregate [18]. Cows are social animals and they readily gather around each other and thus provide an attractive place for stable flies. Our observation suggests that cows often rested near watering places and entrances to feeding lots.

The exclusion of relative humidity caused significant worsening of the model and the interaction term was highly significant, suggesting that humidity is important. It is likely that humid places are preferred during hot summer days [15], which is in accordance with Figure 3, where peak stable fly counts occurred in weeks with very low air humidity (~50%), following very hot weeks. We have no direct data, but it is likely that outside the stable, the air humidity was even lower. Within the stable, stable flies seem to prefer more humid places. As mentioned above, cows bunch together near watering places, where they also breathe, urinate, and make droppings. Thus, places with high cow concentration are likely to be humid. In this respect, the positive effect of humidity may be (at least partly) attributed to the presence of cows.

Conditions within stables are partly protected from outdoor weather, but during hot summer days, the huge inner ventilators are switched on. Strong air flows generated by the ventilators certainly influence the offer of resting places for stable flies [31], as the air flow is strong enough to tear up sheets of paper. However, ventilation of stables is irregular, and is managed by the farm staff. Strong ventilation of the stable provides a possible method to reduce the annoyance of cattle caused by flies. To be effective, it should be applied during the peak stable fly activity in the morning or evening.

A well-known phenomenon of insect behavior is positive phototaxic movement, exploited, for example, by the Malaise trap. Possible aggregations of stable flies might occur near windows, especially because of their small-scale movements. This was supported by our first analysis, as the amount of light has a significant and positive effect on stable fly counts per sticky trap. However, in the second model, there was no significant effect of light, so this relationship remains disputable. As the number of explaining variables was rather high, the inclusion of interaction terms was impractical. High numbers of possible interactions would lead to a complicated, difficult-to-interpret model. Interaction between light conditions, small scale migration of stable flies, and possibly other factors cannot be ruled out.

## 5. Conclusions

In conclusion, it seems that stable flies prefer large congregations of cows; avoid strong air currents; and prefer humid, possibly sunny places. From a practical point of view, two points are important. First, in the relatively warm and dry conditions of south-western Slovakia, the temperature is barely limiting. Differences in stable fly counts were striking in the three study years, but the temperatures were fairly similar. In contrast, the difference in rainfall was substantial and consonant to stable fly traps. High May rainfall thus prognoses high stable fly abundance. Second, the most effective time to reduce stable flies within stables is during hot summer days, when they search for shelter.

## Figures and Tables

**Table 1:** Analysis of variance (ANOVA) table of the final model. The non-significant terms were dropped from the model and the new models were compared with the null model. Dropping of the terms caused significant worsening of the model. Explaining variables of the model: Rain2–6: rainfall two to six weeks before the collecting session, Rain0: rainfall during the session week, Lightness: the amount of light near sticky trap.

Explaining Variable	Degrees of Freedom	F-Value	*p*-Value
Rain0	1	3.44373	0.0637
Temp	1	3.44373	0.0189
I (Temp^2)	1	1 9.78357	0.0018
Lightness	1	6.70538	0.0097
Rain2	1	0.02048	0.8862
Rain3	1	0.36287	0.5470
Rain4	1	15.83749	0.0001
Rain5	1	11.34247	0.0008
Rain6	1	70.05793	<0.0001
Rain0:Temp	1	7.59076	0.0059

**Table 2:** Table of coefficients of the first model, their standard error, *t*-value, and significance.

Explaining Variable	Value	Std. Error	*t*-Value	*p*-Value
Rain0	−0.01700186	0.00702815	−2.419110	0.0157
Temp	0.06144481	0.03687853	1.666140	0.0959
I (Temp^2)	−0.00196213	0.00093244	−2.104301	0.0355
Lightness	0.00047655	0.00019235	2.477555	0.0133
Rain2	0.00716741	0.00231954	3.090012	0.0020
Rain3	0.01343076	0.00271520	4.946501	<0.0001
Rain4	0.02149854	0.00278139	7.729439	<0.0001
Rain5	0.02381396	0.00291648	8.165319	<0.0001
Rain6	0.02197462	0.00255319	8.606741	<0.0001
Rain0:Temp	0.00106967	0.00038704	2.763735	0.0058

**Table 3:** ANOVA table of model of subset of our data from the year 2017. Explaining variables of the model: Meant: mean temperature, Cows: number of cows per stable, Rhmax: maximal humudity, Rain1–6: rainfall one to six weeks before the collecting session, Rain0: rainfall during the session week.

Explaining Variable	Degrees of Freedom	F-Value	*p*-Value
Intercept	1	928.5887	<0.0001
Temp	1	153.4803	<0.0001
I (Temp^2)	1	4.9303	0.0269
Cows	1	2.1380	0.1444
RH	1	0.2558	0.6132
Rain0	1	8.1726	0.0044
Rain1	1	0.3314	0.5651
Rain2	1	7.2047	0.0075
Rain3	1	0.1574	0.6917
Rain4	1	17.4547	<0.0001
Rain5	1	31.0819	<0.0001
Rain6	1	17.1727	<0.0001
Temp: RH	1	17.5873	<0.0001

**Table 4:** Table of coefficients of the second model, their standard error, *t*-value, and significance.

Explaining Variable	Value	Std.Error	*t*-Value	*p*-Value
Intercept	−17.091325	4.087169	−4.181702	<0.0001
Temp	1.164165	0.282152	4.126026	<0.0001
I (Temp^2)	−0.004766	0.002907	−1.639317	0.1018
Cows	0.006042	0.005065	1.192846	0.2335
RH	0.192477	0.048544	3.964992	0.0001
Rain0	0.020426	0.005785	3.531002	0.0005
Rain1	0.018945	0.005866	3.229297	0.0013
Rain2	0.018552	0.005020	3.695718	0.0002
Rain3	0.018162	0.005491	3.307604	0.0010
Rain4	0.016372	0.005232	3.129426	0.0019
Rain5	0.025985	0.005237	4.961893	<0.0001
Rain6	0.026274	0.005382	4.881715	<0.0001
Temp: RH	−0.012419	0.002921	−4.251944	<0.0001
